# Association of Genetic Polymorphisms in Oxidative Stress and Inflammation Pathways with Glaucoma Risk and Phenotype

**DOI:** 10.3390/jcm10051148

**Published:** 2021-03-09

**Authors:** Makedonka Atanasovska Velkovska, Katja Goričar, Tanja Blagus, Vita Dolžan, Barbara Cvenkel

**Affiliations:** 1 Department of Ophthalmology, University Medical Centre Ljubljana, 1000 Ljubljana, Slovenia; atmakedonka@yahoo.com; 2 Pharmacogenetics Laboratory, Institute of Biochemistry and Molecular Genetics, Faculty of Medicine, University of Ljubljana, 1000 Ljubljana, Slovenia; katja.goricar@mf.uni-lj.si (K.G.); tanja.blagus@mf.uni-lj.si (T.B.); vita.dolzan@mf.uni-lj.si (V.D.); 3 Faculty of Medicine, University of Ljubljana, 1000 Ljubljana, Slovenia

**Keywords:** glaucoma, inflammation, oxidative stress, phenotype, polymorphism, susceptibility

## Abstract

Oxidative stress and neuroinflammation are involved in the pathogenesis and progression of glaucoma. Our aim was to evaluate the impact of selected single-nucleotide polymorphisms in inflammation and oxidative stress genes on the risk of glaucoma, the patients’ clinical characteristics and the glaucoma phenotype. In total, 307 patients with primary open-angle glaucoma or ocular hypertension were enrolled. The control group included 339 healthy Slovenian blood donors. DNA was isolated from peripheral blood. Genotyping was performed for *SOD2* rs4880, *CAT* rs1001179, *GPX1* rs1050450, *GSTP1* rs1695, *GSTM1* gene deletion, *GSTT*1 gene deletion, *IL1B* rs1143623, *IL1B* rs16944, *IL6* rs1800795 and *TNF* rs1800629. We found a nominally significant association of *GSTM1* gene deletion with decreased risk of ocular hypertension and a protective role of *IL1B* rs16944 and *IL6* rs1800629 in the risk of glaucoma. The CT and TT genotypes of *GPX1* rs1050450 were significantly associated with advanced disease, lower intraocular pressure and a larger vertical cup–disc ratio. In conclusion, genetic variability in *IL1B* and *IL6* may be associated with glaucoma risk, while *GPX* and *TNF* may be associated with the glaucoma phenotype. In the future, improved knowledge of these pathways has the potential for new strategies and personalised treatment of glaucoma.

## 1. Introduction

There is growing interest in the correlation between oxidative stress, inflammation, apoptosis and primary open-angle glaucoma (POAG) initiation and progression [1,2,3,4,5]. Reactive oxygen species (ROS) are formed in the eyes, following a wide variety of stressors and are largely implicated in glaucoma pathogenesis. Similarly, immune-inflammatory response mediators have recently become a target of interest in glaucoma [6,7,8,9,10].

POAG progression has been linked to an increase in oxidative stress (OS) markers [11,12], and although it has been hypothesised that OS plays an early role in the development of glaucomatous optic neuropathy [13], the link between OS and clinical glaucoma parameters remains to be elucidated [1]. In glaucoma, increased intraocular pressure (IOP), vascular dysregulation and immune system activation can trigger several changes in the retina and optic nerve, including disrupted axonal transport and neurofilament accumulation, microvascular abnormalities, extracellular matrix remodelling and glial cell activation. These alterations can lead to secondary damage such as excitotoxicity, neurotrophin deprivation, oxidative damage, mitochondrial dysfunction and, eventually, retinal ganglion cell death [5,14]. In addition, neurodegeneration extends beyond the retina and optic nerve into the central nervous system [3,15].

Oxidative stress arises due to disturbed equilibrium between the pro-oxidant/antioxidant status that further takes part in the generation of ROS and free radicals, both potentially toxic for neuronal cells. The human body produces oxygen free radicals and other ROS as by-products through numerous physiological and biochemical processes [16]. At the same time, antioxidants, further supported with antioxidant enzymes superoxide dismutase (SOD), catalase (CAT), glutathione reductase and glutathione peroxidase (GPX), help to regulate the level of ROS [17,18]. Decreased antioxidant defence, together with increasing pro-oxidants in the aqueous humour [12,19], ocular tissues [20,21] and blood [22], has been reported in glaucoma. Several antioxidant enzymes such as SOD, CAT and GPX have been found in the aqueous humour [23,24]. The enzyme CAT is the primary scavenger of ROS, and its deficiency and/or genetic variants are associated with a higher risk of diabetes complications and vitiligo [25,26]. GPX has antioxidant effects and catalyses the reduction of hydrogen peroxide by two molecules of glutathione as part of a ROS defence system.

Glaucoma is a neurodegenerative optic neuropathy and, similarly to other neurodegenerative diseases, is also associated with neuroinflammation where astrocytes and microglia play a major role [27]. The presence of reactive astrocytes, microglial activation and the release of inflammatory mediators such as cytokines, ROS, nitric oxide (NO) and tumour necrosis factor-α (TNF-α) cause a state of chronic inflammation that may exert neurotoxic effects [28]. Immune inflammatory response mediators like proteolytic enzymes and pro-inflammatory cytokines (TNF-α, IL-1β, IL-6, IL-12, and NO) have recently become a target of glaucoma research [15,29,30,31,32,33,34,35,36,37,38,39,40]. TNF-α, a major immunomodulator and inflammatory cytokine, has been suggested to mediate the apoptotic death of retinal ganglion cells in glaucoma patients [34]. Interleukin-1, an inflammatory cytokine, is implicated in ischaemic and excitotoxic damage in the retina [41]. Interleukin-6, a proinflammatory cytokine, modulates neuronal survival and protects retinal ganglion cells from ischaemic and excitotoxic damage [42].

Moreover, further research concerning the functions, effectors and signalling pathways of the above molecules and their interactions may lead to new strategies for the treatment of glaucoma [10,43].

Genetic factors, such as single-nucleotide polymorphisms (SNPs), have been shown to modify these pathways, but their impact on the risk of glaucoma and disease course has not been confirmed yet. Studies evaluating the effect of genetic polymorphisms or alterations in proteins’ functions in the oxidative-stress- and inflammation-related genes on the risk of glaucoma have found conflicting results [44,45]. Although the association of genetic variability of inflammation and oxidative stress genes with the risk of glaucoma has already been explored to some extent, the association with patients’ clinical characteristics and glaucoma phenotype has not been studied yet.

Therefore, the aim of our study was to investigate the associations of selected SNPs in inflammation and oxidative stress pathways with the risk of glaucoma, as well as the associations between selected SNPs, patients’ clinical characteristics and the glaucoma phenotype.

## 2. Materials and Methods

### 2.1. Study Participants

The study included patients older than 40 years with POAG or ocular hypertension (OHT) attending the Glaucoma Clinic at the Department of Ophthalmology of the University Medical Centre Ljubljana, Slovenia, from October 2018 until May 2020. The study was approved by the Slovenian Medical Ethics Committee (KME 30/05/11). All subjects signed informed consent in accordance with the Declaration of Helsinki.

Ocular hypertension was defined according to the Guidelines and Terminology of the European Glaucoma Society as IOP higher than 21 mmHg without changes at the optic nerve head and visual field defects [46]. POAG was defined as untreated IOP higher than 21 mmHg, characteristic glaucoma changes at the optic nerve head and/or corresponding visual field defects. Patients with primary juvenile open-angle glaucoma, secondary causes of OHT/glaucoma and non-glaucomatous optic neuropathy and patients after prior intraocular surgery (except non-complicated cataract surgery with more than 6 months after surgery) were excluded. Patients were treated according to the recommendations of the European Glaucoma Society guidelines [47]. Lowering of the IOP was achieved by topical medications, laser trabeculoplasty or surgery, depending on the severity of glaucoma, life expectancy, status of the fellow eye and the rate of progression.

Data on the course of disease and treatment were obtained from medical records. In all patients, the following data were recorded: sex, age, smoking status, a family history of glaucoma, IOP, central corneal thickness (CCT), visual field parameters (with best-corrected visual acuity), vertical cup–disc (C/D) ratio, current ocular diagnosis and systemic diseases. The presence of diabetes, arterial hypertension (AH), hyperlipidaemia and heart disease was also recorded.

The glaucoma phenotype was assessed with the severity of disease, C/D ratio, IOP and CCT. The severity of glaucoma was based on the visual field index mean defect (MD) of the Octopus standard automated perimetry (G program, dynamic strategy) and classified into early (MD < 6 dB), moderate (MD 6–12 dB) and advanced (MD > 12 dB) disease. The IOP was measured by Goldman applanation tonometry. The mean IOP based on all measurements during follow-up was calculated for each eye. CCT values were measured with a manual ultrasound pachymeter (Pachmate-DGH, Tehnology Inc., Exton, PA, USA).

The control group included 339 healthy, unrelated Slovenian blood donors without any systemic disease. For the control group, data about age and sex were available.

### 2.2. DNA Isolation and Genotyping

Nine candidate genes were selected based on their direct involvement in oxidative stress pathways and signalling cascades of inflammation. Only functional polymorphisms with a minor allele frequency above 5% were included in the study: non-synonymous SNPs in the coding region previously associated with enzyme activity or SNPs in the 5’ untranslated region previously associated with altered gene or protein expression levels were *SOD2* rs4880, *CAT* rs1001179, *GPX1* rs1050450, *GSTP1* rs1695, *IL1B* rs1143623, *IL1B* rs16944, *IL6* rs1800795 and *TNF* rs1800629 (Supplementary Materials Table S1) [48]. We also determined the presence of homozygous *GSTM1* and *GSTT1* gene deletion.

Genomic DNA was isolated from peripheral blood samples using the E.Z.N.A.^®^ SQ II Blood DNA Kit (Omega Bio-tek, Inc., Norcross, GA, USA) following the manufacturer’s instructions. *GSTM1* and *GSTT1* were genotyped using multiplex PCR that enabled the detection of homozygous gene deletion. In brief, both genes were simultaneously amplified in a single-step PCR together with the β-globin gene (*HBB*) as the internal positive control, as previously described [49] (Table S2, Figure S1). Genotyping of 8 SNPs was performed with competitive allele-specific PCR (KASP), using the KASP Master mix and custom KASP genotyping assays (all from LGC, Middlesex, UK) according to the manufacturer’s instructions (KBiosciences, Herts, UK and LGC Genomics, UK) (Table S2, Figure S2). Ten percent of the samples were genotyped in duplicate as quality control, and all the results were concordant.

### 2.3. Statistical Analysis

Continuous and categorical variables were described with the median and interquartile range (25–75%) or frequencies, respectively. Fisher’s exact test was used to compare the distribution of categorical variables, while nonparametric Mann–Whitney or Kruskal-Wallis tests were used to compare the distribution of continuous variables. Deviation from the Hardy–Weinberg equilibrium (HWE) was evaluated using the standard chi-square test. Both dominant and additive genetic models were used in the analysis. The association of polymorphisms with glaucoma risk was evaluated using logistic regression to calculate non-adjusted and adjusted odds ratios (ORs) and 95% confidence intervals (CIs). For the IOP, CCT and C/D ratio, data from the most affected eye were used in the analysis. If a measurement was available for only one eye, the measurement of that eye was used in the analysis.

All statistical tests were two sided. As 11 SNPs were investigated, the Bonferroni correction was used to account for multiple comparisons: *p*-values below 0.005 were considered statistically significant, while *p*-values between 0.005 and 0.050 were considered nominally significant. All statistical tests were two sided. In risk analysis, for a polymorphism with a minor allele frequency of 0.10, 0.30 and 0.50, this study had 80% power to detect ORs of 1.9, 1.6 and 1.54 or more, respectively. Power calculation was conducted using PS Power and Sample Size Calculation version 3.0 [50]. The statistical analyses were carried out using IBM SPSS Statistics version 21.0 (IBM Corporation, Armonk, NY, USA).

## 3. Results

A total of 307 patients, 235 with POAG and 72 with OHT, participated in this study. The median age of patients was 70 (interquartile range 64–78) years. Among the patients, 139 (45.3%) were male and 168 (54.7%) were female. The control group consisted of 339 healthy blood donors with the median age of 49 (interquartile range 45–55) years. Among the controls, 251 (74.0%) were male and 88 (26.0%) were female. Patients with POAG or OHT were significantly older than controls (*p* < 0.001). There were more females among patients with POAG or OHT compared to controls (*p* < 0.001). In total, 31.9% of patients reported a family history of glaucoma. Clinical characteristics of all patients, their smoking history and accompanying systemic diseases are presented separately for the OHT and POAG groups in Table 1.

Genotypes’ distributions for the investigated SNPs in both patient and control groups are presented in Table 2. When susceptibility analysis was performed, *GSTM1* gene deletion was nominally significantly associated with the risk for POAG or OHT. The carriers of *GSTM1* gene deletion had lower odds for developing POAG or OHT (OR = 0.50; 95% CI = 0.30–0.83; *p* = 0.007).

Next, susceptibility analysis was performed separately for patients with POAG (*n* = 235) and OHT (*n* = 72), as shown in Table 3. The carriers of *GSTM1* gene deletion had lower odds for developing OHT (OR = 0.43; 95% CI = 0.22–0.81; *p* = 0.009) but not for developing POAG (OR = 0.61; 95% CI = 0.31–1.18; *p* = 0.141).

*IL6* and *IL1B* polymorphisms showed nominally significant association with POAG but not with OHT. The carriers of the *IL1B* rs16944 polymorphism had lower odds for developing POAG in the dominant and additive genetic model (OR = 0.33; 95% CI = 0.13–0.82; *p* = 0.017). *IL6* rs1800795 was associated with lower odds for developing POAG in the dominant genetic model (OR = 0.46; 95% CI = 0.23–0.91; *p* = 0.025) (Table 3).

The distribution of genotype frequencies for the investigated polymorphisms was compared between groups of patients with OHT without visual field defects and patients with glaucoma of different severity (Table 4). The results showed statistically significant differences among groups only for the distribution of *GPX1* rs1050450 genotypes in the dominant model. The carriers of at least one polymorphic *GPX1* allele had less OHT and more POAG (*p* = 0.025; Table 4).

We also analysed the association of the investigated polymorphisms with the glaucoma phenotype, such as the IOP, CCT and C/D ratio (Table 5). The results showed a statistically significant association of *GPX1* rs1050450 with the IOP and C/D ratio in the dominant model. The carriers of at least one polymorphic *GPX1* allele had a lower IOP (*p* = 0.019) and a slightly increased C/D ratio (*p* = 0.035). In addition, a statistically significant association of *TNF* rs*1800629* with the CCT was found in the dominant model. The carriers of at least one polymorphic *TNF* rs*1800629* allele had a larger CCT (*p* = 0.001).

We also analysed the association between patients’ clinical characteristics and the glaucoma phenotype. Cases with AH had a significantly lower maximal median IOP compared to those without AH (*p* < 0.001) and a greater C/D ratio (*p* = 0.008) (Table 6). Similarly, ischaemic heart disease was significantly associated with lower IOP (*p* = 0.002). However, patients with glaucoma were older compared to OHT subjects (*p* < 0.001) with a higher prevalence of heart disease (*p* = 0.034). Diabetes was significantly associated with a larger CCT (*p* = 0.030).

In the multivariable analysis including *GPX1* rs1050450, AH and diabetes, both *GPX1* rs1050450 (*p* = 0.014) and patients’ clinical characteristics remained significantly associated with the IOP (*p* < 0.001 and *p* = 0.019, respectively). In the multivariable analysis including *GPX1* rs1050450, AH and smoking, only *GPX1* rs1050450 remained significantly associated with the C/D ratio (*p* = 0.027). In the multivariable model including *TNF* rs1*800629* and diabetes, both variables were significantly associated with the CCT (*p* < 0.001 and *p* = 0.015).

## 4. Discussion

In this study, we evaluated the associations of selected SNPs in antioxidative and inflammation pathways with the risk of glaucoma and the glaucoma phenotype. Our main finding was that in the antioxidative pathways, *GSTM1* gene deletion may play a protective role in the development of OHT, while inflammatory pathway polymorphisms such as *IL1B* rs16944 and *IL6* rs1800795 may play a protective role in the development of POAG. Interestingly, *GPX1* rs1050450 polymorphism was mainly associated with the severity of POAG and with the phenotype such as the IOP and C/D ratio.

In our study, we observed that the carriers of *GSTM1* gene deletion had lower odds for developing OHT, but we found no association between *GSTT1* gene deletion and the risk of OHT or POAG. GSTs play an important role in cellular protection against oxidative stress. Homozygous *GSTM1* and *GSTT1* deletion results in the absence of the encoded enzymes and may thus impair detoxification and inactivation of reactive metabolites generated during oxidative stress. *GSTM1* and *GSTT1* gene deletion was extensively investigated with regard to glaucoma risk. Whereas some studies have found a significantly higher frequency of the *GSTM1* null genotype in patients with POAG, especially in smokers compared to controls [51,52,53], others have reported increased risk with a *GSTM1* positive phenotype, or in combination with a *GSTT1* null genotype, [54,55] or did not find any association with the risk of glaucoma [56,57,58]. Many factors might account for the differences in results between similar studies, such as differences in sample size, types of glaucoma and ethnic and geographical *GSTM1* null and *GSTT1* null distribution in populations. A recent meta-analysis suggested that there might be a significant association between *GSTM1* polymorphisms and increased susceptibility to glaucoma [57].

In our study, *GPX1* rs1050450 was not associated with the risk of glaucoma. We also found no association between the *CAT* rs1001179 promoter variant and the risk of glaucoma. CAT and GPX1 directly participate in the inactivation of hydrogen peroxide, while GPX1 also participates in the detoxification of reactive secondary metabolites of oxidative stress, such as various lipid hydroperoxides. The most common *GPX1* rs1050450 polymorphism codes for Pro198Leu substitution and leads to decreased enzyme activity; therefore, the capacity for antioxidant defence may also be decreased [59]. Defence capacity against ROS may also be decreased in carriers of a common functional *CAT* rs1001179 polymorphism that influences transcription factor binding in the promoter region and is associated with decreased catalase levels [60]. Associations of *GPX1* polymorphisms have only been studied in other neurodegenerative diseases such as Alzheimer’s disease [61] and Parkinson’s disease [48] but not in glaucoma. Likewise, *CAT* rs1001179 genotype frequencies did not differ significantly between cases and controls in previous studies [24,62]. Only the synonymous SNP *CAT* rs769217 was significantly associated with POAG in the Chinese population [62]. We also found no association between *SOD* rs4880 and glaucoma. Similarly, no difference in the allele and genotype frequency in SNPs rs4880 between POAG cases and controls was reported by Zhou et al. [63].

With regard to inflammatory pathway polymorphisms, we found a protective effect of *IL1B* rs16944 and *IL6* rs1800795 on the development of POAG. Both polymorphisms are located in the promoter region and may lead to altered gene expression. In patients with POAG, significantly increased mRNA expression of the *IL1B* gene has been found in blood and significantly increased IL-1B protein expression in the aqueous humour compared to controls [64]. In an animal model of acute glaucoma, upregulation of IL-1B caused an increase in retinal ganglion cell death [65]. Polymorphisms in the *IL1B* promoter region (rs16944 and rs1143634—not analysed in our study) have already been investigated for an association with POAG. Whereas positive associations were reported in Caucasian populations [66,67], associations with POAG were not observed in Asian populations [68,69,70,71]. Meta-analysis evaluating the role of these two SNPs of *IL1B* in the susceptibility to glaucoma did not find any association, but the conclusions should be interpreted with caution as only a small number of studies was included [72].

With regard to *IL6*, Zimmermann et al. suggested that the promoter *IL6 rs1800795* polymorphism is unlikely to be a major risk factor for POAG [73]. In addition, a recent systematic review and meta-analysis found a statistically significant glaucoma risk associated only with rs1524107, but not with rs1800795, which was investigated in our study [42]. However, when patients with early to moderate glaucoma were compared to patients with advanced glaucoma, the *IL6* rs1800795 C allele as well as the GC genotype were protective against less severe forms of normal-tension glaucoma [69].

We are not aware of any studies investigating the association of SNPs involved in the inflammatory and oxidative stress pathways with the glaucoma phenotype, including the severity of glaucoma, IOP, C/D ratio and CCT. We found a statistically significant association of *GPX1* rs1050450 with the severity of glaucoma. In the dominant model, the frequency of at least one polymorphic *GPX* allele increased with the severity of glaucoma (19.3% in early, 23.3% in moderate and 39.3% in advanced glaucoma). The *GPX1* rs1050450 CT and TT genotypes were reported to be associated with increased risk of damage caused by oxidative stress, such as in coronary heart disease and cancer [74,75]. The *GPX1* rs1050450 CT and TT genotypes were associated with increased risk of POAG in the Polish population, but the link with glaucoma phenotypes has not been investigated [76]. Antioxidant enzymes, such as GPX, are an important defence system against oxidative stress, which may play a major pathophysiological role in glaucoma. An increase in oxidative stress markers in serum and aqueous humour with a decrease in serum antioxidant stress markers was present in glaucoma patients compared to controls. However, despite a decrease in serum GPX, there was an important increase in GPX in the aqueous humour [11]. This may indicate a protective response of the eye against oxidative stress and may wear off in the long term [19].

Glaucoma is a heritable disease, and siblings of POAG cases have a tenfold-increased risk of developing the disease [77]. The C/D ratio, IOP and CCT used clinically to predict POAG risk are heritable traits related to the disease and may be associated with genetic variability in inflammation and oxidative stress pathways. In our study, carriers of at least one polymorphic *GPX1* rs1050450 allele had statistically lower IOP and a lower C/D ratio. Patients with advanced glaucoma require lower target IOP to prevent progression of disease than those with early glaucoma or ocular hypertension [78]. In our study, approximately 30% of all cases had advanced disease. Therefore, those with lower IOP had presumably advanced glaucoma, which might partly explain the link between *GPX1* polymorphisms and lower IOP and severity of glaucoma. However, the association between this SNP and the C/D ratio is not clear. We found no previous studies analysing the association of the *GPX1* SNP with the glaucoma phenotype.

In the last decade, genome-wide association studies (GWAS) have identified over 50 C/D-ratio-associated loci, but only 9 of these have been associated with POAG [79,80]. Up till now, multiple IOP-associated loci were identified using large and multi-ethnic biobank-based cohorts. Among the significant results were also loci at genes previously associated with POAG but not previously known to influence IOP. This indicates that genetic variation at these genes mediates the increased POAG risk via raised IOP rather than via direct effect on retinal ganglion cells [79,80]. The identified loci explained 17% of the variance of IOP in the EPIC–Norfolk Eye Study [81].

The central corneal thickness has been associated with increased POAG development and progression [82,83], but it is uncertain whether this relationship is caused by IOP measurement artefacts or whether the relationship is biologically causal [84]. In our study, the GG genotype of *TNF* rs1800629 was associated with a low CCT. The link between *TNF* polymorphisms and the CCT has not been investigated. However, recently, the results of GWAS for the CCT have suggested that the CCT may not be a heritable trait for POAG and that the CCT–glaucoma association observed in studies is due to IOP measurement artefacts rather than biological causality [80].

Multiple epidemiological studies have reported the role of hypertension as a risk factor for POAG [85,86]. Treatment of hypertensive patients with beta-blockers results in nocturnal hypotension, which is a potential risk factor for glaucomatous optic neuropathy [87,88]. The mechanisms by which hypertension induces optic nerve damage are still unclear. Whether or not an association exists between diabetes mellitus and glaucoma has been an issue of debate, but findings from several studies in recent years suggest that the risk of glaucoma among diabetic patients may be greater than once believed [89,90,91,92,93]. Patients with POAG may suffer from ischaemic heart disease more often than those without glaucoma [93]. We also analysed the association between clinical characteristics (AH, diabetes, ischaemic heart disease, family history of glaucoma) with the glaucoma phenotype (IOP, C/D ratio, CCT). In our study, patients with AH had significantly lower maximal median IOP and a greater C/D ratio compared to those without AH. A possible explanation is that among cases with AH, there were older patients with advanced glaucoma, requiring lower target IOP compared to cases with OHT. Similarly, in our study, patients with ischaemic heart disease had significantly lower IOP. As patients with glaucoma were older compared to OHT subjects and with a higher prevalence of heart disease, they required lower target IOP to prevent progression, which could explain our results. Diabetes was significantly associated with a larger CCT in our study. Our observation is in line with the findings of a meta-analysis that suggested that diabetes and hyperglycaemia are associated with a thicker cornea [94]. We found no other associations between the glaucoma phenotype or clinical characteristics with the investigated SNPs.

One of the limitations of our study was that the number of control subjects and cases was small compared to larger studies investigating genetic factors. Furthermore, we investigated only a limited number of polymorphisms in the oxidative stress and inflammation pathways. The strength of our study was that we used a pathway-based approach and selected SNPs with a known functional effect that are common in the Caucasian population. Perhaps other polymorphisms of the genes involved in these pathways, not investigated in our study, may have had a potential impact. Another limitation was that only data on age and sex were available for the control group. However, all controls were healthy blood donors without any self-reported systemic disease. Furthermore, the chance of controls having undiagnosed glaucoma was small due to the low prevalence of the disease in this age group, estimated to be between 0.2% and 1.1% in a Caucasian population of the same age [95,96,97]. Another strength of our study was that all patients attended the same glaucoma unit with the same treatment approach and follow-up, unambiguous diagnostic criteria and classification of phenotype. Furthermore, all patients and controls originated from a genetically homogenous population, thus limiting possible bias due to the population structure [98,99].

Although our findings should be interpreted with caution, there is further perspective to this research. Ganglion cell death in glaucoma is a complex process triggered by different molecular mechanisms, among which oxidative stress and activation of inflammation by retinal glial cells play an important role. Improving antioxidant defence and addressing inflammation pathways might stimulate cell survival and boost the cells’ ability to withstand pathological insult. Several studies have shown the potential protective effect of antioxidants on retinal ganglion cells [100,101,102,103], while there is experimental evidence that modulation of inflammation reduces retinal ganglion cell death [102]. Therefore, improved knowledge of these pathways might help to establish predictive biomarkers to improve treatment strategies for glaucoma. Patients could be stratified into groups with detectable deficits in oxidative stress and/or inflammation pathways, so supplementary therapy could be more specific and treatment personalised. Our study investigated genes and SNPs with broad implications in glaucoma and other neurodegenerative diseases that share similar biomarkers [104,105]. This type of study on glaucoma and similar diseases may help to design inflammation and oxidative stress pathway gene panels that could be used in testing patients with different but related diseases in order to personalise treatment and potentially improve treatment outcomes.

In conclusion, we used a pathway-based approach to address the relationship between oxidative stress and inflammation polymorphisms, and POAG risk. We found some indications for a possible association of genetic variability in *GSTM1* with OHT. While *IL1B* and *IL6* may be associated with the risk of glaucoma, *GPX* and *TNF* may affect the glaucoma phenotype. However, the evidence presented here is limited and further association and functional studies are required.

## Figures and Tables

**Table 1:** Clinical characteristics of patients.

Characteristic		Cases (*n* = 307)	OHT (*n* = 72)	POAG (*n* = 235)	*p*-Value
Sex	Male, *n* (%)	139 (45.3)	34 (47.2)	105 (44.7)	0.787
	Female, *n* (%)	168 (54.7)	38 (52.8)	130 (55.3)	
Age (years)	Median (25–75%)	70 (64–78)	64 (54–69)	72 (66–79)	<0.001
IOP (mmHg)	Right eye, (median (25–75%)	19.71 (16.8–23)	22.6 (20.1–24.2)	18.59 (16.3–22.0)	<0.001
	Left eye, (median (25–75%)	19.78 (16.7–23)	22.5 (19.7–24.2)	18.48 (16.3–22.0)	<0.001
CCT (µm)	Right eye, (median (25–75%)	547 (521–574)	565.5 (533–595)	541 (518–570)	<0.001
	Left eye, (median (25–75%)	548.5 (522.5–577.0) {1}	570.5 (532.3–602.5)	543 (519.5–571.0)	<0.001
C/D ratio	Right eye, (median (25–75%)	0.8 (0.5–0.9)	0.35 (0.3–0.5)	0.9 (0.7–1.0)	<0.001
	Left eye, (median (25–75%)	0.8 (0.5–1)	0.3 (0.3–0.5)	0.9 (0.7–1.0)	<0.001
Family history of glaucoma	No, *n* (%)	209 (68.1)	51 (70.8)	158 (67.2)	0.665
	Yes, *n* (%)	98 (31.9)	21 (29.2)	77 (32.8)	
Arterial hypertension {1}	No, *n* (%)	140 (45.8)	38 (52.8%)	102 (43.6)	0.179
	Yes, *n* (%)	166 (54.2)	34 (47.2%)	132 (56.4)	
Diabetes	No, *n* (%)	280 (91.2)	64 (88.9%)	216 (91.9)	0.476
	Yes, *n* (%)	27 (8.8)	8 (11.1%)	19 (8.1)	
Hyperlipidaemia	No, *n* (%)	209 (68.1)	53 (73.6%)	156 (66.4)	0.312
	Yes, *n* (%)	98 (31.9)	19 (26.4%)	79 (3.6)	
Heart disease {1}	No, *n* (%)	240 (78.4)	63 (87.5%)	177 (75.6)	0.034
	Yes, *n* (%)	66 (21.6)	9 (12.5%)	57 (24.4)	
Smoking	No, *n* (%)	204 (66.4)	40 (55.6%)	164 (69.8)	0.045
	Currently, *n* (%)	26 (8.5)	6 (8.3)	20 (8.5)	
	Former, *n* (%)	77 (25.1)	26 (36.1)	51 (21.7)	

{ }—missing data; OHT—ocular hypertension; POAG—primary open-angle glaucoma; IOP—intraocular pressure; CCT—central corneal thickness; C/D ratio—vertical cup–disc ratio.

**Table 2:** Genotype frequencies of the control (*n* = 339) and patient (*n* = 307) groups with the risk of glaucoma or OHT.

SNP	Genotype	Controls *n* (%)	Cases *n* (%)	OR (95% CI) adj.	*p*_adj_-Value
*SOD2* rs4880	CC	92 (27.1)	78 (25.4)	Reference	
	CT	160 (47.2)	136 (44.3)	1.11 (0.60–2.06)	0.745
	TT	87 (25.7)	93 (30.3)	1.15 (0.58–2.26)	0.690
	CT + TT	247 (72.9)	229 (74.6)	1.12 (0.63–2.00)	0.691
*CAT* rs1001179	CC	193 (57.1) {1}	184 (59.9)	Reference	
	CT	122 (36.1)	105 (34.2)	0.95 (0.56–1.60)	0.840
	TT	23 (6.8)	18 (5.9)	1.31 (0.42–4.08)	0.642
	CT + TT	145 (42.9)	123 (40.1)	0.99 (0.59–1.63)	0.954
*GPX1* rs1050450	CC	170 (50.1)	157 (51.1)	Reference	
	CT	130 (38.3)	125 (40.7)	0.86 (0.51–1.47)	0.592
	TT	39 (11.5)	25 (8.1)	0.49 (0.19–1.28)	0.146
	CT + TT	169 (49.9)	150 (48.9)	0.78 (0.47–1.30)	0.345
*GSTP1* rs1695	AA	150 (44.2)	141 (45.9)	Reference	
	AG	152 (44.8)	128 (41.7)	0.98 (0.58–1.67)	0.940
	GG	37 (10.9)	38 (12.4)	1.84 (0.79–4.28)	0.157
	AG + GG	189 (55.8)	166 (54.1)	1.11 (0.67–1.83)	0.686
*GSTP1* rs1138272	CC	274 (80.8)	254 (82.7)	Reference	
	CT + TT	65 (19.2)	53 (17.3)	1.25 (0.65–2.41)	0.513
*GSTM1* gene deletion	No deletion	136 (40.1)	150 (48.9)	Reference	
	Deletion	203 (59.9)	157 (51.1)	0.50 (0.30–0.83)	**0.007**
*GSTT1* gene deletion	No deletion	288 (85)	254 (82.7)	Reference	
	Deletion	51 (15)	53 (17.3)	0.58 (0.30–1.12)	0.103
*IL1B* rs1143623	GG	174 (51.3)	145 (47.2)	Reference	
	GC	136 (40.1)	135 (44)	1.25 (0.74–2.11)	0.408
	CC	29 (8.6)	27 (8.8)	1.50 (0.61–3.69)	0.378
	GC + CC	165 (48.7)	162 (52.8)	1.29 (0.78–2.12)	0.323
*IL1B* rs16944	TT	44 (13)	44 (14.3)	Reference	
	TC	145 (42.8)	143 (46.6)	0.69 (0.32–1.47)	0.332
	CC	150 (44.2)	120 (39.1)	0.62 (0.28–1.35)	0.227
	TC + CC	295 (87.0)	263 (85.7)	0.65 (0.32–1.34)	0.248
*IL6* rs1800795	GG	120 (35.4)	111 (36.2)	Reference	
	GC	151 (44.5)	154 (50.2)	0.71 (0.41–1.24)	0.226
	CC	68 (20.1)	42 (13.7)	0.67 (0.32–1.39)	0.277
	GC + CC	219 (64.6)	196 (63.8)	0.70 (0.41–1.17)	0.174
*TNF* rs1800629	GG	228 (67.3)	234 (76.2)	Reference	
	GA + AA	111 (32.7)	73 (23.8)	0.66 (0.38–1.17)	0.156

{ }—missing data; adj–adjusted for age and sex; SNP–single nucleotide polymorphism. Nominally significant values have been marked in bold.

**Table 3:** Genotype frequencies of selected polymorphisms and their association with the risk of OHT (*n* = 72) and POAG (*n* = 235).

		OHT			POAG		
SNP	Genotype	*n* (%)	OR (95% CI) adj.	*p*_adj_-Value	*n* (%)	OR (95% CI) adj.	*p*_adj_-Value
*SOD2* rs4880	CC	15 (20.8)	Reference		63 (26.8)	Reference	
	CT	39 (54.2)	1.37 (0.63–3.00)	0.426	97 (41.3)	0.90 (0.41–2.02)	0.806
	TT	18 (25)	1.12 (0.46–2.71)	0.798	75 (31.9)	1.23 (0.51–2.93)	0.643
	CT + TT	57 (79.2)	1.28 (0.61–2.66)	0.515	172 (73.2)	1.02 (0.49–2.14)	0.953
*CAT* rs1001179	CC	49 (68.1)	Reference		135 (57.4)	Reference	
	CT	20 (27.8)	0.66 (0.33–1.30)	0.230	85 (36.2)	1.36 (0.68–2.69)	0.381
	TT	3 (4.2)	0.40 (0.07–2.19)	0.290	15 (6.4)	3.79 (0.92–15.55)	0.064
	CT + TT	23 (31.9)	0.62 (0.32–1.20)	0.157	100 (42.6)	1.54 (0.80–2.96)	0.201
*GPX1* rs1050450	CC	45 (62.5)	Reference		112 (47.7)	Reference	
	CT	22 (30.6)	0.66 (0.33–1.30)	0.228	103 (43.8)	0.93 (0.47–1.85)	0.834
	TT	5 (6.9)	0.36 (0.10–1.26)	0.111	20 (8.5)	0.70 (0.20–2.44)	0.581
	CT + TT	27 (37.5)	0.59 (0.31–1.12)	0.104	123 (52.3)	0.89 (0.46–1.72)	0.725
*GSTP1* rs1695	AA	38 (52.8)	Reference		103 (43.8)	Reference	
	AG	26 (36.1)	0.92 (0.47–1.79)	0.800	102 (43.4)	0.92 (0.46–1.84)	0.819
	GG	8 (11.1)	1.37 (0.49–3.83)	0.554	30 (12.8)	2.70 (0.85–8.60)	0.092
	AG + GG	34 (47.2)	0.99 (0.53–1.86)	0.987	132 (56.2)	1.11 (0.58–2.13)	0.747
*GSTP1* rs1138272	CC	62 (86.1)	Reference		192 (81.7)	Reference	
	CT + TT	10 (13.9)	1.09 (0.47–2.55)	0.837	43 (18.3)	1.44 (0.60–3.47)	0.410
*GSTM1* gene deletion	No deletion	36 (50)	Reference		114 (48.5)	Reference	
	Deletion	36 (50)	0.43 (0.22–0.81)	**0.009**	121 (51.5)	0.61 (0.31–1.18)	0.141
*GSTT1* gene deletion	No deletion	60 (83.3)	Reference		194 (82.6)	Reference	
	Deletion	12 (16.7)	0.61 (0.26–1.44)	0.258	41 (17.4)	0.50 (0.22–1.16)	0.107
*IL1B* rs1143623	GG	38 (52.8)	Reference		107 (45.5)	Reference	
	GC	32 (44.4)	1.16 (0.61–2.19)	0.657	103 (43.8)	1.12 (0.56–2.24)	0.745
	CC	2 (2.8)	0.47 (0.10–2.24)	0.341	25 (10.6)	2.87 (0.95–8.65)	0.061
	GC + CC	34 (47.2)	1.04 (0.56–1.95)	0.893	128 (54.5)	1.34 (0.70–2.57)	0.384
*IL1B* rs16944	TT	5 (6.9)	Reference		39 (16.6)	Reference	
	TC	34 (47.2)	1.38 (0.45–4.28)	0.574	109 (46.4)	0.34 (0.13–0.90)	**0.030**
	CC	33 (45.8)	1.44 (0.46–4.47)	0.533	87 (37)	0.32 (0.12–0.86)	**0.024**
	TC + CC	67 (93.1)	1.41 (0.48–4.16)	0.536	196 (83.4)	0.33 (0.13–0.82)	**0.017**
*IL6* rs1800795	GG	25 (34.7)	Reference		86 (36.6)	Reference	
	GC	36 (50)	0.89 (0.45–1.80)	0.755	118 (50.2)	0.44 (0.21–0.93)	**0.031**
	CC	11 (15.3)	0.77 (0.30–1.96)	0.577	31 (13.2)	0.49 (0.19–1.29)	0.149
	GC + CC	47 (65.3)	0.86 (0.44–1.66)	0.654	149 (63.4)	0.46 (0.23–0.91)	**0.025**
*TNF* rs1800629	GG	56 (77.8)	Reference		178 (75.7)	Reference	
	GA + AA	16 (22.2)	0.62 (0.30–1.28)	0.196	57 (24.3)	0.67 (0.31–1.41)	0.289

adj—adjusted for age and sex; OHT—ocular hypertension; POAG—primary open-angle glaucoma; SNP—single nucleotide polymorphism. Nominally significant values have been marked in bold.

**Table 4:** Association of selected polymorphisms with OHT and POAG severity.

SNP	Genotype	OHT (*n* = 72)	Early POAG (*n* = 62)	Moderate POAG (*n* = 54)	Severe POAG (*n* = 119)	*p*-Value *
		*n* (%)	*n* (%)	*n* (%)	*n* (%)	
*SOD2* rs4880	CC	15 (19.2)	15 (19.2)	15 (19.2)	33 (42.3)	Padd = 0.681
	CT	39 (28.7)	27 (19.9)	22 (16.2)	48 (35.3)	
	TT	18 (19.4)	20 (21.5)	17 (18.3)	38 (40.9)	
	CT + TT	57 (24.9)	47 (20.5)	39 (17)	86 (37.6)	Pdom = 0.722
*CAT* rs1001179	CC	49 (26.6)	31 (16.8)	35 (19)	69 (37.5)	Padd = 0.149
	CT	20 (19)	23 (21.9)	18 (17.1)	44 (41.9)	
	TT	3 (16.7)	8 (44.4)	1 (5.6)	6 (33.3)	
	CT + TT	23 (18.7)	31 (25.2)	19 (15.4)	50 (40.7)	Pdom = 0.158
*GPX1* rs1050450	CC	45 (28.7)	33 (21)	19 (12.1)	60 (38.2)	Padd = 0.134
	CT	22 (17.6)	24 (19.2)	29 (23.2)	50 (40)	
	TT	5 (20)	5 (20)	6 (24)	9 (36)	
	CT + TT	27 (18)	29 (19.3)	35 (23.3)	59 (39.3)	**Pdom = 0.025**
*GSTP1* rs1695	AA	38 (27)	26 (18.4)	23 (16.3)	54 (38.3)	Padd = 0.628
	AG	26 (20.3)	30 (23.4)	21 (16.4)	51 (39.8)	
	GG	8 (21.1)	6 (15.8)	10 (26.3)	14 (36.8)	
	AG + GG	34 (20.5)	36 (21.7)	31 (18.7)	65 (39.2)	Pdom = 0.570
*GSTP1* rs1138272	CC	62 (24.4)	49 (19.3)	41 (16.1)	102 (40.2)	0.299
	CT + TT	10 (18.9)	13 (24.5)	13 (24.5)	17 (32.1)	
*GSTM1* gene deletion	No deletion	36 (24)	23 (15.3)	30 (20)	61 (40.7)	0.195
	Deletion	36 (22.9)	39 (24.8)	24 (15.3)	58 (36.9)	
*GSTT*1 gene deletion	No deletion	60 (23.6)	50 (19.7)	46 (18.1)	98 (38.6)	0.940
	Deletion	12 (22.6)	12 (22.6)	8 (15.1)	21 (39.6)	
*IL1B* rs1143623	GG	38 (26.2)	27 (18.6)	22 (15.2)	58 (40)	Padd = 0.358
	GC	32 (23.7)	27 (20)	26 (19.3)	50 (37)	
	CC	2 (7.4)	8 (29.6)	6 (22.2)	11 (40.7)	
	GC + CC	34 (21)	35 (21.6)	32 (19.8)	61 (37.7)	Pdom = 0.526
*IL1B* rs16944	TT	5 (11.4)	14 (31.8)	7 (15.9)	18 (40.9)	Padd = 0.074
	TC	34 (23.8)	30 (21)	30 (21)	49 (34.3)	
	CC	33 (27.5)	18 (15)	17 (14.2)	52 (43.3)	
	TC + CC	67 (25.5)	48 (18.3)	47 (17.9)	101 (38.4)	Pdom = 0.079
*IL6* rs1800795	GG	25 (22.5)	24 (21.6)	23 (20.7)	39 (35.1)	Padd = 0.862
	GC	36 (23.4)	31 (20.1)	23 (14.9)	64 (41.6)	
	CC	11 (26.2)	7 (16.7)	8 (19)	16 (38.1)	
	GC + CC	47 (24)	38 (19.4)	31 (15.8)	80 (40.8)	Pdom = 0.617
*TNF* rs1800629	GG	56 (23.9)	46 (19.7)	43 (18.4)	89 (38)	0.873
	GA + AA	16 (21.9)	16 (21.9)	11 (15.1)	30 (41.1)	

* Comparison of all 4 groups using Fisher’s exact test; add—additive model; dom—dominant model; OHT—ocular hypertension; POAG—primary open-angle glaucoma; SNP—single nucleotide polymorphism. Statistically significant values have been marked in bold.

**Table 5:** Association of investigated polymorphisms with the IOP, CCT and C/D ratio.

SNP	Genotype	IOP Max Median (25–75%)	*p*-Value	CCT Min. Median (25–75%)	*p*-Value	C/D Max. Median (25–75%)	*p*-Value
*SOD2* rs4880	CC	21 (17.5–26)	Padd = 0.301	539 (520.75–571)	Padd = 0.422	0.85 (0.6–1)	Padd = 0.246
	CT	20.8 (17.5–23.3)		548 (520–577)		0.8 (0.5–1)	
	TT	20.2 (17.3–23)		541 (514.5–573)		0.9 (0.7–1)	
	CT + TT	20.6 (17.5–23)	Pdom = 0.139	544 (518.5–573)	Pdom = 0.590	0.9 (0.6–1)	Pdom = 0.985
*CAT* rs1001179	CC	20.8 (17.5–23.7)	Padd = 0.574	543 (518.25–573.75)	Padd = 0.331	0.85 (0.6–1)	Padd = 0.517
	CT	20.6 (17.4–23.7)		541 (519.5–571.5)		0.9 (0.6–1)	
	TT	22.5 (17.5–26)		557 (532.5–584.5)		0.8 (0.6–0.9)	
	CT + TT	20.8 (17.5–24)	Pdom = 0.759	544 (520–573)	Pdom = 0.994	0.9 (0.6–1)	Pdom = 0.690
*GPX1* rs1050450	CC	21.2 (17.9–24)	Padd = 0.061	551 (520.5–575)	Padd = 0.245	0.8 (0.5–1)	Padd = 0.105
	CT	19.4 (17.2–23)		538 (519–571)		0.9 (0.7–1)	
	TT	19 (17.2–24)		540 (501.5–572)		0.9 (0.6–1)	
	CT + TT	19.3 (17.2–23.2)	**Pdom = 0.019**	538.5 (518–571)	Pdom = 0.094	0.9 (0.675–1)	**Pdom = 0.035**
*GSTP1* rs1695	AA	20.5 (17.6–23.5)	Padd = 0.991	546 (519.5–575)	Padd = 0.154	0.8 (0.55–1)	Padd = 0.572
	AG	21 (17.2–24)		537.5 (515.5–570)		0.9 (0.6–1)	
	GG	20.1 (17.7–24.1)		547.5 (531–574.75)		0.9 (0.7–1)	
	AG + GG	20.9 (17.4–24)	Pdom = 0.899	540.5 (520–572.25)	Pdom = 0.598	0.9 (0.6–1)	Pdom = 0.357
*GSTP1* rs1138272	CC	20.6 (17.5–24)	0.924	544 (520–573)	0.965	0.9 (0.6–1)	0.846
	CT + TT	21 (17.4–23.7)		540 (519.5–575.5)		0.9 (0.6–1)	
*GSTM1* gene deletion	No deletion	21 (17.2–24)	0.967	544 (515–576)	0.894	0.9 (0.6–1)	0.823
	Deletion	20.6 (17.6–23.5)		541 (520–572.5)		0.8 (0.6–1)	
*GSTT1* gene deletion	No deletion	20.7 (17.4–23.9)	0.354	541.5 (518–573)	0.199	0.9 (0.6–1)	0.994
	Deletion	21 (18–24)		544 (529.5–577)		0.9 (0.6–1)	
*IL1B* rs1143623	GG	21 (17.6–24)	Padd = 0.268	539 (518–575)	Padd = 0.382	0.9 (0.6–1)	Padd = 0.843
	GC	20.2 (17.2–23)		544 (520–571)		0.8 (0.6–1)	
	CC	21 (17.6–24)		557 (530–573)		0.9 (0.6–1)	
	GC + CC	20.3 (17.3–23)	Pdom = 0.128	547 (521–571.5)	Pdom = 0.383	0.85 (0.6–1)	Pdom = 0.970
*IL1B* rs16944	TT	20.4 (17.7–23.1)	Padd = 0.411	551 (529.25–570)	Padd = 0.617	0.9 (0.7–1)	Padd = 0.541
	TC	20.4 (17.5–23)		544 (520–574)		0.8 (0.6–1)	
	CC	21 (17.2–25)		538.5 (518–573.75)		0.9 (0.5–1)	
	TC + CC	20.8 (17.4–24)	Pdom = 0.989	541 (519–574)	Pdom = 0.508	0.9 (0.6–1)	Pdom = 0.319
*IL6* rs1800795	GG	20 (17.6–23.3)	Padd = 0.184	546 (520–578)	Padd = 0.354	0.8 (0.5–1)	Padd = 0.342
	GC	20.6 (17.2–23.4)		541.5 (520.75–573)		0.9 (0.6–1)	
	CC	21.8 (18.4–26)		534 (502.75–569.25)		0.8 (0.4–1)	
	GC + CC	21 (17.3–24)	Pdom = 0.491	540.5 (518–571)	Pdom = 0.390	0.9 (0.6–1)	Pdom = 0.451
*TNF* rs1800629	GG	20.5 (17.2–23.2)	0.166	540 (517.75–570)	**0.001**	0.9 (0.6–1)	0.539
	GA + AA	21.7 (17.7–24.1)		564 (529.5–589.5)		0.9 (0.6–1)	

add—additive model; dom—dominant model; IOP—intraocular pressure; CCT—central corneal thickness; C/D ratio—vertical cup–disc ratio; SNP—single nucleotide polymorphism. Statistically significant values have been marked in bold.

**Table 6:** Association of patients’ clinical characteristics with the glaucoma phenotype.

Characteristic		IOP Max. Median (25–75%)	*p*-Value	CCT Min. Median (25–75%)	*p*-Value	C/D Ratio Max. Median (25–75%)	*p*-Value
Smoking	No, *n* (%)	20.1 (17.5–23.7)	0.356	541 (520–573)	0.589	0.9 (0.6–1)	**0.019**
	Yes, *n* (%)	21 (17.4–24)		544 (520–573)		0.8 (0.5–1)	
Family history	No, *n* (%)	21 (17.7–24)	0.073	544 (520–574)	0.632	0.9 (0.6–1)	0.695
	Yes, *n* (%)	20 (17.1–23.2)		540.5 (518.8–570.5)		0.9 (0.6–1)	
AH	No, *n* (%)	22 (18.6–24.2)	**<0.001**	548.5 (526.8–575.5)	0.050	0.8 (0.5–1)	**0.008**
	Yes, *n* (%)	19.4 (16.9–22.8)		538 (512–570.3)		0.9 (0.6–1)	
Diabetes	No, *n* (%)	20.6 (17.2–23.7)	0.071	541 (518.3–573)	**0.030**	0.9 (0.6–1)	0.543
	Yes, *n* (%)	22 (19.3–24)		558 (540–580)		0.9 (0.4–1)	
Hyperlipidaemia	No, *n* (%)	21 (17.6–24)	0.078	543 (518.5–574)	0.959	0.8 (0.6–1)	0.541
	Yes, *n* (%)	19.7 (17.2–23.1)		543.5 (520–570)		0.9 (0.6–1)	
Heart disease	No, *n* (%)	21 (17.7–24)	**0.002**	544.5 (520–575.5)	0.173	0.8 (0.6–1)	0.485
	Yes, *n* (%)	19 (16.7–21.5)		536.5 (515.8–562.3)		0.9 (0.6–1)	

AH—arterial hypertension; IOP—intraocular pressure; CCT—central corneal thickness; C/D ratio—vertical cup–disc ratio. Statistically significant values have been marked in bold.

## Data Availability

The datasets used and/or analysed for the current study are available from the corresponding author on reasonable request.

## Supplementary Materials

The following are available online at https://www.mdpi.com/2077-0383/10/5/1148/s1, Table S1: Characteristics of investigated polymorphisms, variant allele frequency and agreement with Hardy-Weinberg equilibrium in controls, Table S2: Primers used for multiplex PCR (a) and thermal cycling conditions used for genotyping for multiplex PCR (b) and KASP chemistry (c), Figure S1: Representative gel image of *GSTT1* and *GSTM1* genotyping analysis, Figure S2: Representative cluster image for *IL6* rs1800795 analysis obtained after KASP competitive allele specific PCR. References [106,107,108,109,110,111] are cited in the supplementary materials.
