# Supplementary material for: Association of Genetic Polymorphisms in Oxidative Stress and Inflammation Pathways with Glaucoma Risk and Phenotype
Source: J Clin Med. 2021 Mar 9;10(5):1148. doi: 10.3390/jcm10051148 (PMC7967191; doi:10.3390/jcm10051148)
Supplement: Supplementary file 1 [file jcm-10-01148-s001.pdf]

Table S1. Characteristics of investigated polymorphisms, variant allele frequency and agreement with Hardy-Weinberg equilibrium in controls.

SNP	Nucleotide/ amino acid change	Location	SNP function	VAF (controls)	*p*HWE (controls)
SOD2 rs4880	p.Ala16Val	Coding region nsSNP	Decreased enzyme activity [1]	0.493	0.304
CAT rs1001179	c.-262C > T	5′UTR	Altered expression [2]	0.249	0.536
GPX1 rs1050450	p.Pro198Leu	Coding region nsSNP	Decreased enzyme activity [3]	0.307	0.070
GSTP1 rs1695	p.Ile105Val	Coding region nsSNP	Decreased enzyme activity [4]	0.333	0.871
GSTP1 rs1138272	p.Ala114Val	Coding region nsSNP	Decreased enzyme activity [4]	0.100	0.805
IL1B rs1143623	c.-1560G > C	5′UTR	Altered expression [5]	0.286	0.741
IL1B rs16944	c.-598T > C	5′UTR	Altered expression [6]	0.656	0.340
IL6 rs1800795	c.-174G > C	5′UTR	Altered expression [7]	0.423	0.106
TNF rs1800629	c.-308 G > A	5′UTR	Altered expression [8]	0.177	0.546

HWE–Hardy-Weinberg equilibrium, ns–non-synonymous, SNP–single nucleotide polymorphism, UTR–untranslated region, VAF–variant allele frequency.

Table S2. Primers used for multiplex PCR (**a**) and thermal cycling conditions used for genotyping for multiplex PCR (**b**) and KASP chemistry (**c**).

**a).**	**Primer sequences**		
GSTM1	Forward 5' CTGGATTGTAGCAGATCATGC 3'		
	Reverse 5' CTCCTGATTATGACAGAAGCC 3'		
GSTT1	Forward 5' TTCCTTACTGGTCCTCACATCTC 3'		
	Reverse 5' TCACCGGATCATGGCCAGCA 3'		
ß-globin	Forward 5' GAAGAGCCAAGGACAGGTAC 3'		
	Reverse 5' CAACTTCATCCACGTTCACC 3'		
**b)**	**Multiplex PCR protocol**		
Stage	Temperature	Duration	Cycles no.
Start denaturation	94 °C	10 min	1
Denaturation	94 °C	30 s	35
Annealing	60 °C	30 s	
Extension	72 °C	30 s	
Final extension	72 °C	10 min	1
**c)**	**61-55 °C Touchdown protocol**		
Stage	Temperature	Duration	Cycles no.
Hot-start Taq activation	94°C	15 min	1
Touchdown	94°C	20 s	10
	61 °C (61 °C decreasing 0.6 °C per cycle to achieve a final annealing / extension temperature of 55 °C)	60 s	
Amplification	94 °C	20 s	30
	55 °C	60 s	
Read stage	30 °C	60 s	1

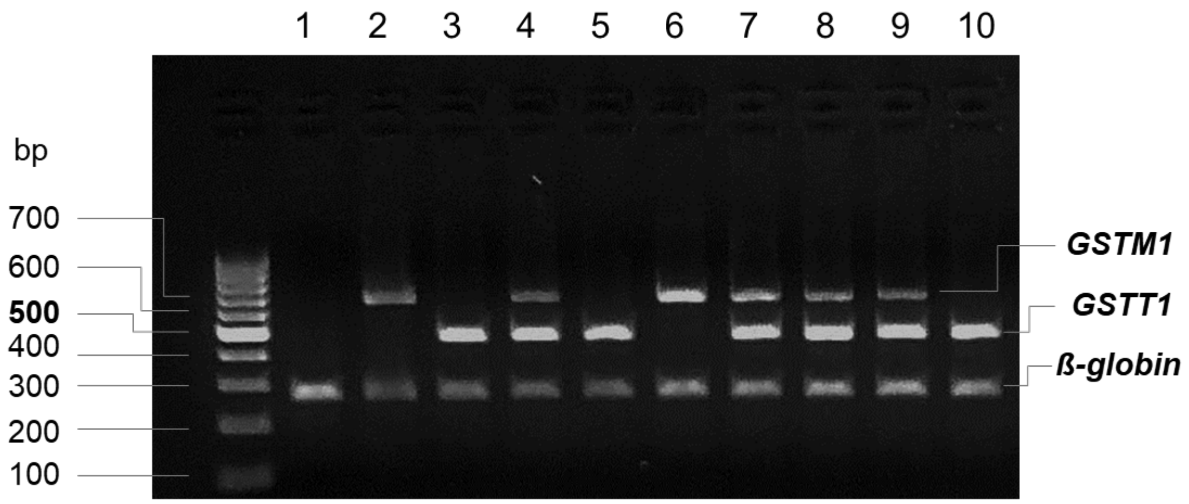

Figure S1. Representative gel image of *GSTT1* and *GSTM1* genotyping analysis. *GSTT1* and/or *GSTM1* deletions were determined simultaneously with multiplex PCR followed by electrophoresis (40 min at 100 V) on 2% agarose gel in TBE buffer stained with ethidium bromide. *GSTM1* and *GSTT1* genotypes were determined by the presence or absence of the respective amplification products. ß-globin served as internal control of amplification. Amplicon lengths: *GSTM1* - 600 bp, *GSTT1* - 480 bp, ß-globin gene - 268 bp. Lane 1: homozygous *GSTM1* and *GSTT1* deletion, lanes 2 and 6: homozygous *GSTT1* deletion, lanes 3, 5 and 10: homozygous *GSTM1* deletion, lanes 4, 7, 8, and 9: *GSTM1* and *GSTT1* gene present. The 100 bp DNA Ladder (Fermentas, Thermo Fisher Sicentific, Waltham, MA, USA) was used as a reference.

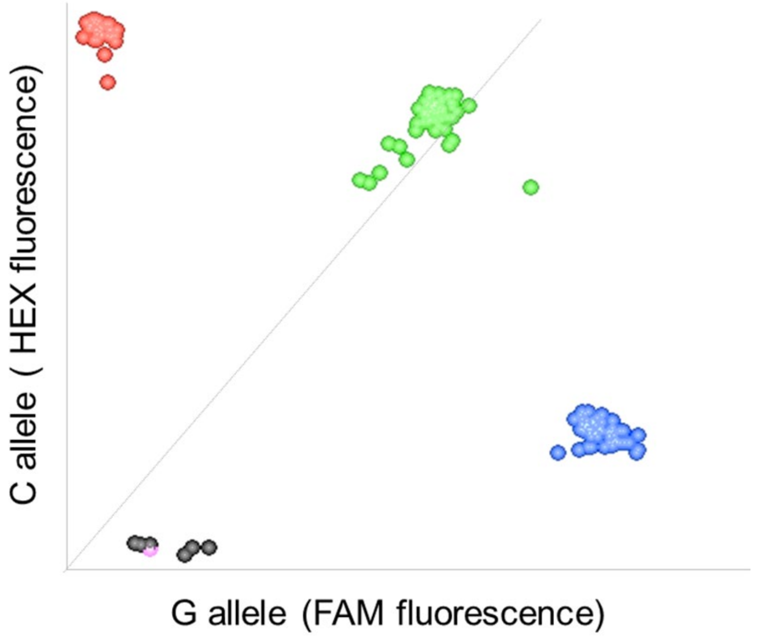

Figure S2. Representative cluster image for *IL6* rs1800795 analysis obtained after KASP competitive allele specific PCR. Fluorescence of amplified products was measured with microplate reader (FLUOstar Omega, BTG LABTECH, Ortenberg, Germany) and processed with KlusterCaller program (LGC Genomics, Hoddesdon, UK). Genotypes were determined considering fluorescence signal detected; for homozygous samples either FAM (labelled G allele) or HEX (labelled C allele) fluorescence signal was detected and for heterozygous both of fluorescence signals were detected. Black cluster: no template controls (NTC); red cluster: homozygous for allele C (CC genotype); green cluster: heterozygous (CG genotype); blue cluster: homozygous for allele G (GG genotype).

References

1. Sutton, A.; Imbert, A.; Igoudjil, A.; Descatoire, V.; Cazanave, S.; Pessayre, D.; Degoul, F. The manganese superoxide dismutase Ala16Val dimorphism modulates both mitochondrial import and mRNA stability. Pharmacogenet Genomics 2005, 15, 311-319, doi:10.1097/01213011-200505000-00006.
2. Forsberg, L.; Lyrenas, L.; de Faire, U.; Morgenstern, R. A common functional C-T substitution polymorphism in the promoter region of the human catalase gene influences transcription factor binding, reporter gene transcription and is correlated to blood catalase levels. Free Radic. Biol. Med. 2001, 30, 500-505.
3. Ravn-Haren, G.; Olsen, A.; Tjønneland, A.; Dragsted, L.O.; Nexø, B.A.; Wallin, H.; Overvad, K.; Raaschou-Nielsen, O.; Vogel, U. Associations between GPX1 Pro198Leu polymorphism, erythrocyte GPX activity, alcohol consumption and breast cancer risk in a prospective cohort study. Carcinogenesis 2006, 27, 820-825, doi:10.1093/carcin/bgi267.
4. Nebert, D.W.; Vasiliou, V. Analysis of the glutathione S-transferase (GST) gene family. Hum Genomics 2004, 1, 460-464, doi:10.1186/1479-7364-1-6-460.
5. Kutikhin, A.G.; Yuzhalin, A.E.; Volkov, A.N.; Zhivotovskiy, A.S.; Brusina, E.B. Correlation between genetic polymorphisms within IL-1B and TLR4 genes and cancer risk in a Russian population: a case-control study. Tumour Biol 2014, 35, 4821-4830.
6. Torres-Merino, S.; Moreno-Sandoval, H.N.; Thompson-Bonilla, M.D.R.; Leon, J.A.O.; Gomez-Conde, E.; Leon-Chavez, B.A.; Martinez-Fong, D.; Gonzalez-Barrios, J.A. Association Between rs3833912/rs16944 SNPs and Risk for Cerebral Palsy in Mexican Children. Mol Neurobiol 2018, 21, 018-1178.
7. Lagmay, J.P.; London, W.B.; Gross, T.G.; Termuhlen, A.; Sullivan, N.; Axel, A.; Mundy, B.; Ranalli, M.; Canner, J.; McGrady, P., et al. Prognostic significance of interleukin-6 single nucleotide polymorphism genotypes in neuroblastoma: rs1800795 (promoter) and rs8192284 (receptor). Clin Cancer Res 2009, 15, 5234-5239.
8. Szkup, M.; Chelmecka, E.; Lubkowska, A.; Owczarek, A.J.; Grochans, E. The influence of the TNFalpha rs1800629 polymorphism on some inflammatory biomarkers in 45-60-year-old women with metabolic syndrome. Aging 2018, 10, 2935-2943.
